# Economic burden of Thalassemia treatment: An analysis from the Vietnam Social Security perspective

**DOI:** 10.1371/journal.pone.0293916

**Published:** 2023-11-27

**Authors:** Hoang-Thy Nhac-Vu, Van Thi-Ngoc Tran, Trong-Duy-Thuc Nguyen, Vu-Thanh Pham, Tho Le

**Affiliations:** 1 Faculty of Pharmacy, University of Medicine and Pharmacy at Ho Chi Minh City, Ho Chi Minh City, Vietnam; 2 Faculty of Medicine, Can Tho University of Medicine and Pharmacy, Can Tho City, Vietnam; 3 General Planning Department, Lam-Dong General Hospital, Da Lat, Lam Dong Province, Vietnam; 4 Lam-Dong Children’s Hospital, Da Lat, Lam Dong Province, Vietnam; Murdoch University, AUSTRALIA

## Abstract

**Objectives:**

Thalassemia is a genetic disorder that significantly impacts the health and well-being of individuals in Vietnam. This study aimed to assess the economic burden of Thalassemia treatment in Lam-Dong Province from the perspective of the Vietnam Social Security and to develop a model to forecast these costs.

**Methods:**

This study analyzed the medical records of all 288 health-insured Thalassemia patients who received treatment in Lam-Dong Province from 2019–2021. The annual economic burden was calculated as the total direct medical cost of treatment per patient over one year. Bayesian Model Averaging (BMA) was utilized to forecast economic burdens. The best fit model was selected based on evaluation criteria including the R2 value, the Bayesian information criterion (BIC), and posterior model probabilities.

**Results:**

The study found that the average annual economic burden of Thalassemia treatment was VND 9,947,000 (±6,854,000), equivalent to approximately USD 426.7 (±294.0), with blood transfusions being the main contributor to costs (63%). Using BMA, the best fit model to forecast economic burdens included variables including patient age, sex, and length of hospitalization, with age being the key factor with the greatest impact on the increase in economic burden.

**Conclusion:**

These findings provided important information for policymakers in Vietnam, as they highlighted the significant economic burden of Thalassemia treatment in the country. By developing a model to forecast these costs, policymakers can make informed decisions on how to allocate resources and support individuals with Thalassemia and their families.

## Introduction

Thalassemia, a genetic disorder characterized by congenital hemolytic anemia, is a common global health concern, with high prevalence rates in the Mediterranean, Middle East, Asia, and Pacific regions [1]. The two major subtypes of Thalassemia, α and β, are caused by mutations in the HBA and HBB genes, respectively. Of these, α-Thalassemia is particularly prevalent in Vietnam, with a reported prevalence rate of 51.5%, making it the highest in Southeast Asia [2]. Thalassemia can result in mild to moderate chronic hemolytic anemia, which can lead to organ damage and reduced lifespan in affected individuals.

The disease is present in all regions of Vietnam, but is more prevalent among mountainous ethnic minorities than among the Kinh ethnicity [3]. Lam Dong province locates in The Central Highlands region of Vietnam and is home to a diverse population that includes the Kinh ethnicity and various other ethnic groups such as K’Ho, Ma, Nung, and Tay. In 2021, the average income in Lam-Dong Province was VND 3.7 million/month, lower than the national average of VND 4.2 million/month [4].

Patients with Thalassemia often require routine blood transfusions to survive, and those with Thalassemia major are at higher risk for premature mortality [5, 6]. The treatment of Thalassemia can be costly and requires ongoing maintenance, leading to economic burdens for patients, their families, and society. To date, there have been no studies examining the economic burden of Thalassemia treatment in Vietnam, particularly in low-income, high-prevalence areas like Lam-Dong Province.

### Conceptual framework

To comprehend the economic burden of Thalassemia treatment in Vietnam, we developed a conceptual framework that considered different factors that could influence the cost of treating Thalassemia. This framework was constructed using existing research and our own observations, with a significant emphasis on patient characteristics:

- Age: The intensity of treatment might differ across ages, with older patients potentially having more complex needs [7];
- Sex: Gender-specific medical needs or biases in treatment can influence costs [8];
- Ethnicity: This socio-demographic variable might affect treatment accessibility and compliance [9];
- Length of hospitalization: Extended stays could increase the overall cost and may reflect the severity of the disease [10].
- Type of Thalassemia: Different types like alpha, beta, HbH, intermedia, or homozygous mutations could have distinct treatment needs and associated costs [11–13].

This conceptual framework was used to guide our empirical analysis and the interpretation of our findings.

### Objectives

This study aimed to assess the economic burden of Thalassemia treatment and develop an empirical model to forecast these costs from the perspective of the Vietnam Social Security (VSS). This information is necessary for informed policy decision-making on behalf of these vulnerable populations.

## Materials and methods

This cross-sectional, descriptive study employed a retrospective design utilizing medical records of social health insured (SHI) Thalassemia patients (ICD-10 D56) of all ages who received treatment in Lam-Dong Province, Vietnam, for a minimum of one year from January 1, 2019, to December 31, 2021. Data were obtained from the Lam-Dong Social Security and three hospitals that provided Thalassemia treatment within the province. After excluding cases with incomplete or insufficient information, a total of 288 cases were eligible for analysis using the whole sampling technique.

This study collected data on direct medical costs, including examination, blood transfusion, laboratory tests, hospitalization days (bed-days), medical supplies, drugs, and diagnostic imaging. The costs were measured in VND (1,000,000 VND was approximately equal to 42.9 USD). Patient characteristics, including age, sex, ethnicity, length of hospitalization, and Thalassemia subtypes, were also collected.

All data were analyzed using R statistical software version 4.1.3. The annual economic burden was calculated as the total direct medical cost of treatment per patient over a one-year period. These costs were calculated in 2022 SHI cost units and were described using statistical measures including mean, standard deviation, median, minimum and maximum values, and visualizations including histogram plots and boxplots. Comparisons of total direct medical costs across patient characteristics were performed using the Mann-Whitney test, with statistical significance defined as p<0.05.

To forecast economic burdens, the empirical Bayesian Model Averaging (BMA) approach was implemented. The best-fit model was selected based on evaluation criteria including the R2 value, the Bayesian information criterion (BIC), and posterior model probabilities. All costs were converted to the logarithm base e before analysis, and outliers were identified and eliminated.

## Results

### Characteristics of Thalassemia patients in Lam-Dong Province, Vietnam

**Fig 1:**
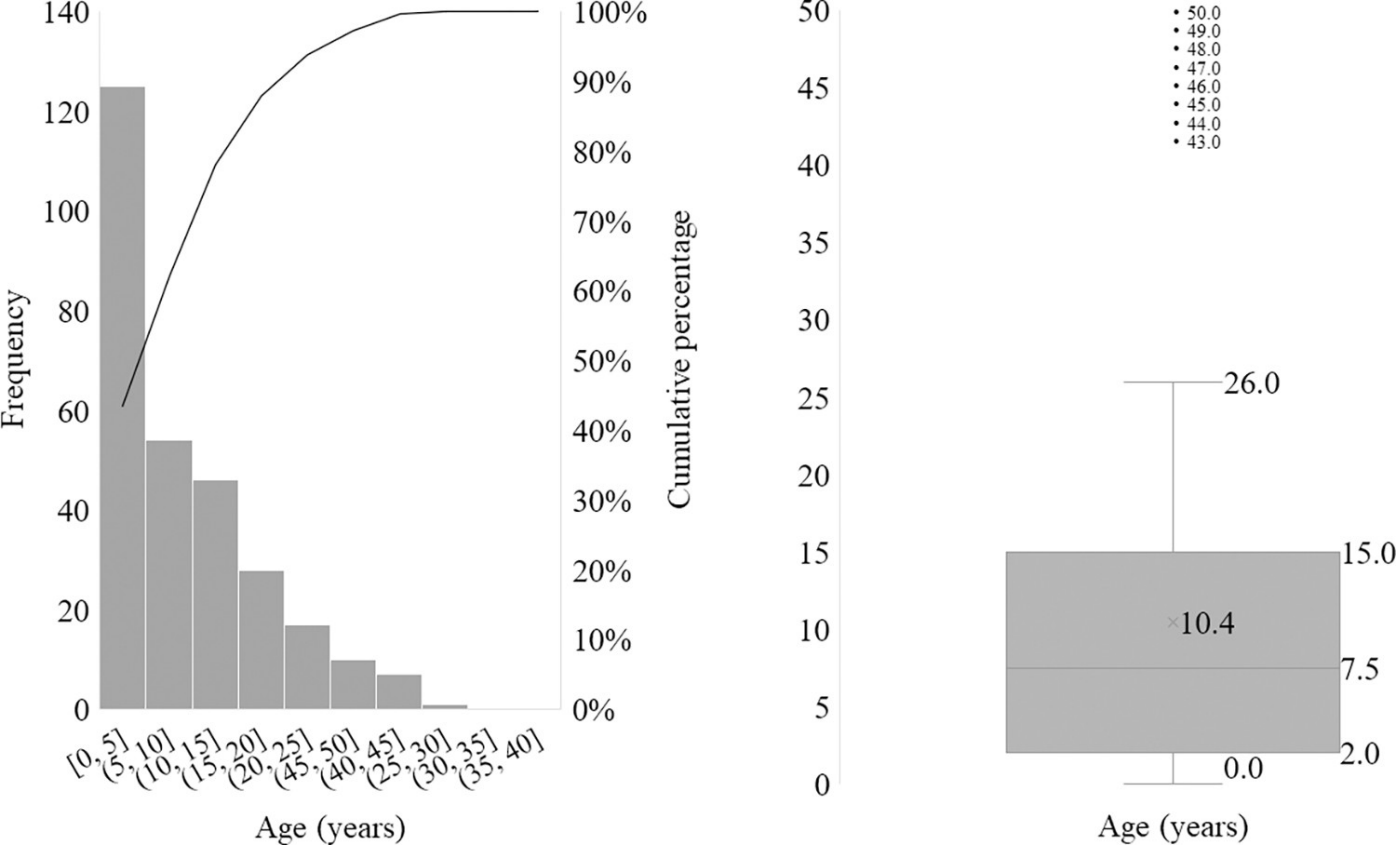
Age distribution of Thalassemia patients in Lam-Dong Province, Vietnam.

In this study, 288 health-insured patients with Thalassemia were analyzed to determine the economic burden of treatment. The sample had a mean age of 10.4 (±11.3) years, with 85.1% being ethnic Kinh and 56.2% being male. The mean length of hospitalization was 7.4 (±6.0) days, with 58.7% of patients being hospitalized for less than 7 days (Fig 1).

### Economic burdens of Thalassemia treatment

**Fig 2:**
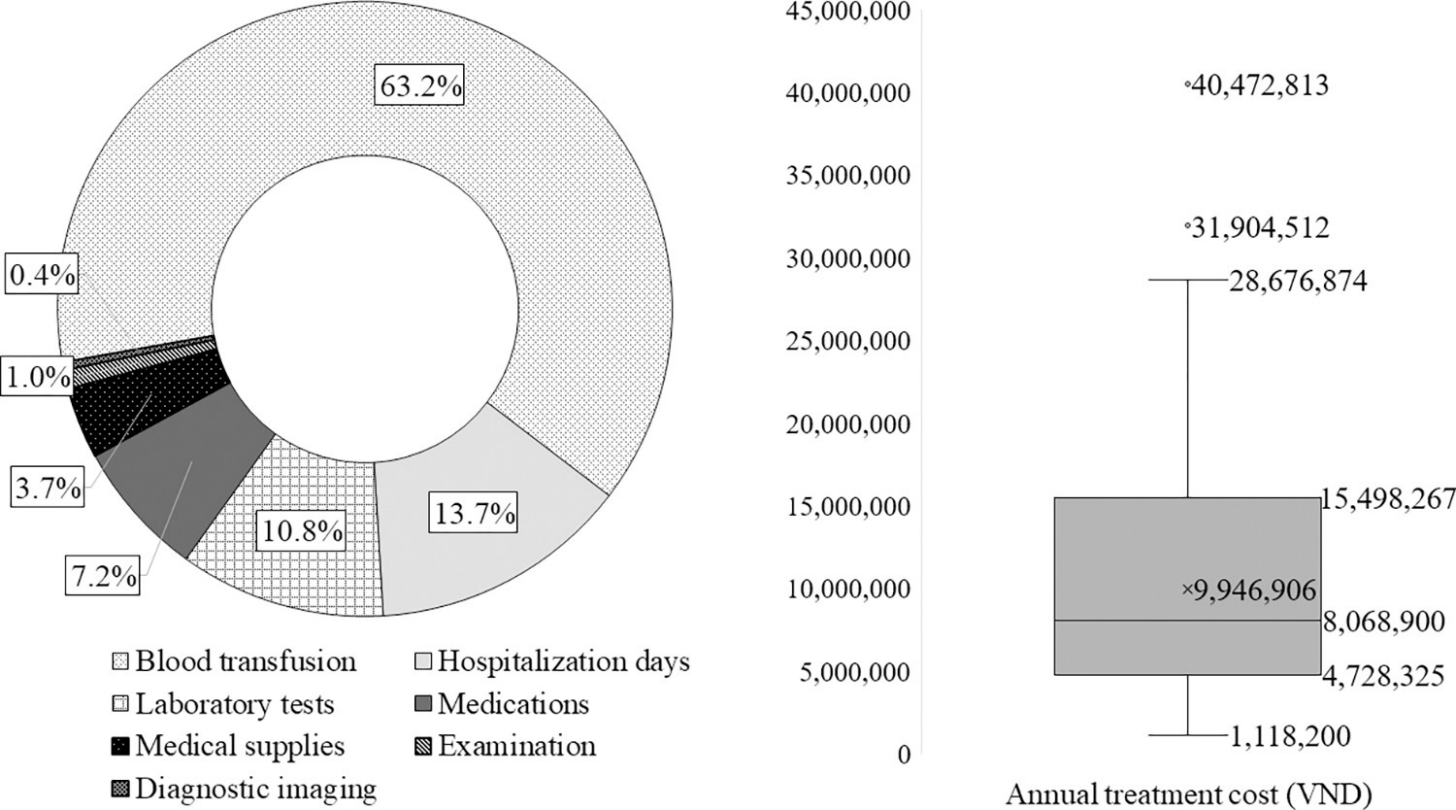
Annual direct medical costs for Thalassemia treatment per patient (VND).

The annual total direct medical costs ranged from VND 1,118,000 to VND 40,473,000 with a mean value of VND 9,947,000 (±6,854,000). Transfusions and hospitalization accounted for the largest portion of costs (63.2% and 13.7%, respectively), while diagnostic imaging had the smallest portion (0.4%) (Fig 2).

There were statistically significant differences in the economic burden of treatment among different patient characteristics, including age, length of hospitalization, and Thalassemia subtypes (p<0.05) (Table 1).

**Table 1:** Annual economic burden for Thalassemia treatment by patient characteristics.

Patient characteristics	Sample n = 288 *(%)*	Annual economic burden per patient (million VND)		p-value
		Mean *(±SD)*	Median *(min-max)*	
**Age (years)**				
<6	125 *(43*.*4)*	8.487 *(±5*.*657)*	6.807 *(1*.*118–26*.*122)*	<0.001
6–10	54 *(18*.*7)*	7.092 *(±5*.*779)*	4.971 *(1*.*137–25*.*758)*	
11–15	46 *(16*.*0)*	11.563 *(±8*.*612)*	8.612 *(1*.*129–40*.*473)*	
≥16	63 *(21*.*9)*	14.111 *(±6*.*334)*	14.061 *(1*.*408–26*.*518)*	
**Sex**				
Male	162 *(56*.*2)*	9.110 *(±5*.*952)*	7.735 *(1*.*118–26*.*591)*	0.116
Female	126 *(43*.*8)*	11.023 *(±7*.*756)*	9.434 *(1*.*232–40*.*473)*	
**Hospitalization days**				
<7	169 *(58*.*7)*	5.494 *(±3*.*232)*	5.122 *(1*.*118–18*.*613)*	<0.001
≥7	119 *(41*.*3)*	16.271 *(±5*.*536)*	16.018 *(5*.*824–40*.*473)*	
**Ethnicity**				
Kinh	245 *(85*.*1)*	9.787 *(±6*.*460)*	8.073 *(1*.*118–26*.*591)*	0.915
Minority	43 *(14*.*9)*	10.861 *(±8*.*808)*	7.784 *(1*.*432–40*.*473)*	
**Thalassemia subtypes 1**				
α	132 *(45*.*8)*	7.063 *(±5*.*450)*	6.042 *(1*.*129–40*.*473)*	<0.001
β	156 *(54*.*2)*	12.387 *(±6*.*986)*	12.771 *(1*.*118–28*.*677)*	
**Thalassemia subtypes 2**				
HbH	132 *(45*.*8)*	7.063 *(±5*.*450)*	6.042 *(1*.*129–40*.*473)*	<0.001
Intermedia	84 *(29*.*2)*	9.526 *(±7*.*127)*	8.011 *(1*.*118–28*.*677)*	
Homozygous mutation	72 *(25*.*0)*	15.724 *(±5*.*118)*	16.407 *(2*.*949–26*.*591)*	

### Bayesian model averaging to forecast economic burden

**Fig 3:**
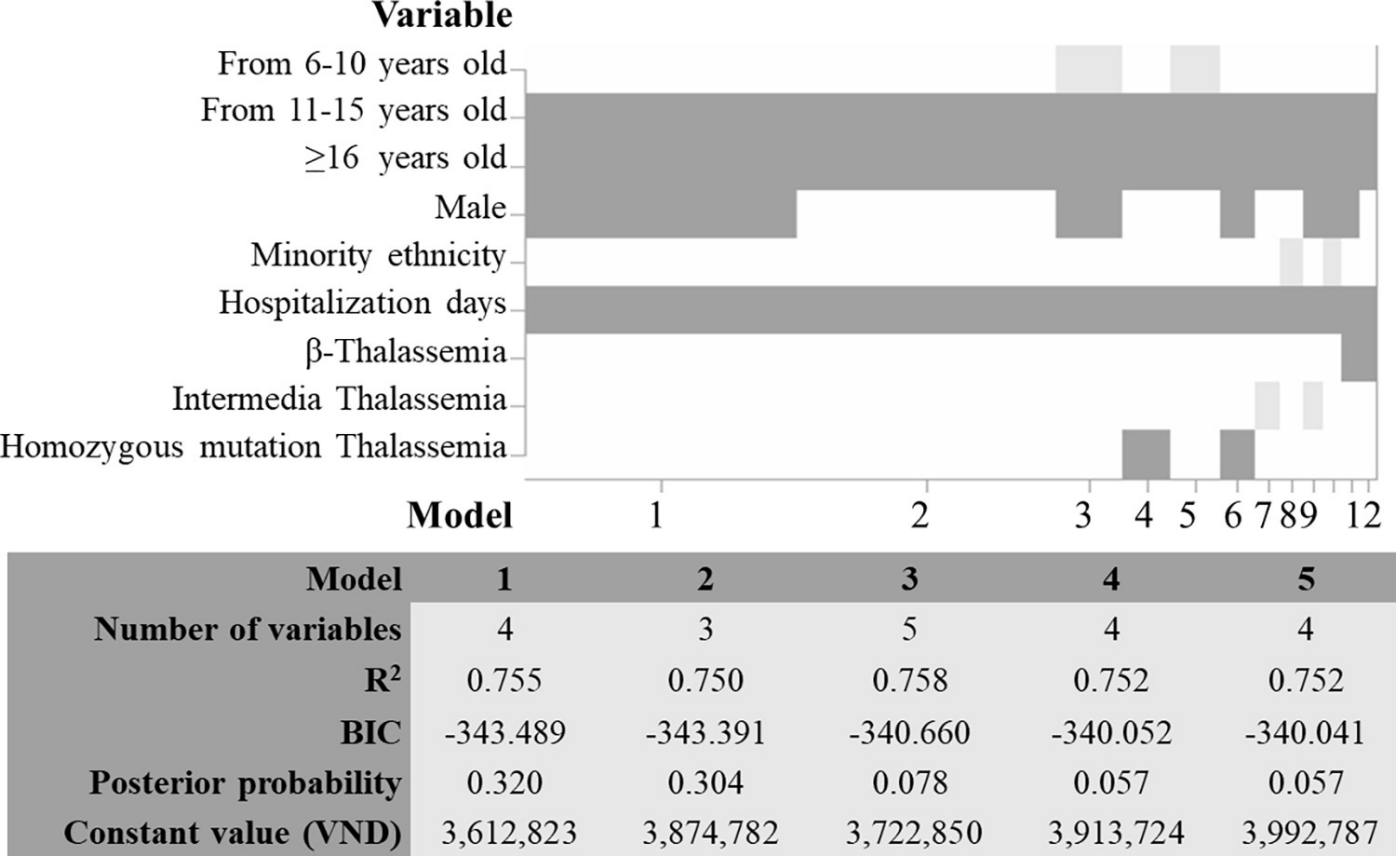
Evaluation of potential models for forecasting economic burden using BMA.

A total of 12 models were established. Of these, the best-fit model was Model 1, which had the lowest Bayesian information criterion (BIC) value and the highest posterior model probability. This model included variables including age, sex, and length of hospitalization, and was represented by the following equation: *Annual Thalassemia economic burden (VND) = exp[15*.*10+ 0*.*26 x (Age | 11–15 years) + 0*.*43 x (Age | ≥16 years) +0*.*10 x (Sex | Male) + 0*.*09 x (Hospitalization days)]*. Factors that were found to increase the economic burden included being aged 11 years or older, being male, and having a longer hospitalization. For example, a female Thalassemia patient younger than 11 years and not hospitalized was forecasted to have an economic burden of VND 3,612,823 (Fig 3).

Age was the key variable that had the greatest impact on the increase in economic burden in all models. In Model 1, treatment costs for patients aged 16 years or older were forecasted to be VND 1,941,000 (53.7%) higher than those for patients aged less than 6 years (Table 2).

**Table 2:** Annual economic burden of Thalassemia treatment: BMA forecasts.

Covariate	Bayesian Model Averaging								
	p≠0	Mean	SD	Model 1 (best fit)	Model 2	Model 3	Model 4	Model 5	Incremental cost (VND)
Constant	100.0	15.14	0.054	15.10	15.17	15.13	15.18	15.20	-
Age: 6–10 *vs* <6 years	13.5	-0.01	0.04	-	-	-0.09	-	-0.08	-
Age: 11–15 *vs* <6 years	100.0	0.26	0.06	0.26	0.26	0.23	0.27	0.24	1,073,000
Age: ≥16 *vs* <6 years	100.0	0.42	0.05	0.43	0.41	0.40	0.44	0.39	1,941,000
Sex: male *vs* female	50.5	0.05	0.06	0.10	-	0.10	-	-	380,000
Ethnicity: minority ethnic *vs* Kinh	4.9	-0.002	0.02	-	-	-	-	-	-
Hospitalization days	100.0	0.09	0.004	0.09	0.09	0.09	0.08	-	340,000
Subtypes 1: β *vs* α	9.7	0.001	0.01	-	-	-	-	-	-
Subtypes 2: intermediate *vs* HbH	5.3	-0.002	0.02	-	-	-	-	-	-
Subtypes 2: homozygous mutation *vs* HbH	9.8	-0.009	0.03	-	-	-	0.10	-	-

## Discussion

This study found that treatment for Thalassemia was financially burdensome for low-income patients in Vietnam. Currently, the VSS is playing an important role in supporting Thalassemia patients by providing the SHI scheme which covers 100% for children under 6 years old or for the poor and near poor and 80% for the rest. Without the SHI, treatment for Thalassemia would cost nearly a quarter of an adult’s annual income in Lam-Dong Province, not yet to mention indirect costs such as pain, distress, or premature death caused by Thalassemia, which would be unaffordable for many patients and could lead to poverty [4]. In addition, 25% of the patients in this study had major Thalassemia, a severe form of the condition with a shortened lifespan. Therefore, a health insurance scheme with full insurance coverage for Thalassemia medications and expanding subsidies for supportive care may help patients and their families cope with financial hardship and mental distress, as well as to ensure healthcare equity within the society.

Compared to previous research, this study found similar treatment cost structures, but with significantly different mean costs [14, 15]. Blood transfusion was the main cost component in this study (accounting for 63.2% of total costs). Iron chelation drugs, which were often prescribed to treat iron poisoning from multiple blood transfusions, also made up a significant proportion of Thalassemia treatment costs. In 2021, at the Lam-Dong Children’s Hospital, two iron chelation drugs, desferrioxamine IV and deferasirox IV, accounted for 85% of the total cost of medicine for thalassemia treatment. The mean cost per year in this study (VND 9.95 million VND or USD 426.7) was lower than in other countries such as Thailand (USD 1,080), Greece (USD 26,736), Turkey (USD 25,832), and Iran (USD 29,593), but higher than in India (USD 329.6) [16–20]. These differences might be due to variations in unit costs, social health insurance policies, cost components, and patient characteristics between studies.

This study also developed a model to forecast the annual economic burden of Thalassemia treatment from the VSS perspective. Factors associated with the annual economic burden included length of hospitalization (longer stays were associated with higher costs), sex (males had higher costs), and age (patients aged 11 years or older had higher costs). These factors were considered to be reasonable, as older Thalassemia patients with longer hospital stays would require more medical services. While treatment costs did vary based on Thalassemia subtypes, these factors were not included in the best-fit model, which differed from previous studies [16, 21].

While the results of this study were novel and representative, being based on data collected over three consecutive years in the entire Lam-Dong Province and considering all aspects of direct medical costs covered by the VSS, it is important to acknowledge its limitations. The study was restricted by the availability of electronic data for only the past three years and the absence of patient-level data, such as iron chelation drug costs, which may introduce bias. The study focused solely on Vietnam, particularly investigating the economic impact of Thalassemia, and making a basic comparison with similar studies from other countries. To overcome these limitations, future studies can broaden the scope by conducting a comprehensive cross-country comparative analysis and using data spanning a longer time frame. This approach would provide a more complete and well-rounded understanding of the subject. Nevertheless, this study stands as the first to utilize Bayesian model averaging to forecast the economic burden of Thalassemia, providing higher quality evidence for policymakers in Vietnam to comprehend the cost of Thalassemia treatment and make informed decisions on insurance policies.

## Conclusion

This study is the first to examine the economic burden and develop a model to forecast Thalassemia treatment costs in Lam-Dong Province, Vietnam. The treatment was costly for low-income patients as it cost nearly a quarter of an adult’s annual income in the region. Policymakers can leverage these insights to consider alternative treatments with lower costs and extend SHI coverage to alleviate the burden on patients and ensure healthcare equity.
